# A Multi‐Scale Computational Framework for Anti‐Persister Tuberculosis Drug Discovery

**DOI:** 10.1111/cbdd.70408

**Published:** 2026-10-03

**Authors:** Said Moshawih, Anguraj Moulishankar, Chee Yan Choo, Khang Wen Goh, Chrismawan Ardianto, Azam Che Idris, Long Chiau Ming

**Affiliations:** ^1^ Faculty of Pharmacy, Department of Pharmaceutical Sciences Al‐Ahliyya Amman University Amman Jordan; ^2^ Faculty of Data Science and Information Technology INTI International University Nilai Malaysia; ^3^ Department of Pharmaceutical Chemistry, Integrated Drug Discovery Centre Acharya & BM Reddy College of Pharmacy Bengaluru Karnataka India; ^4^ Faculty of Pharmacy Universiti Teknologi MARA Shah Alam Selangor Malaysia; ^5^ Faculty of Pharmacy Universitas Airlangga Surabaya Indonesia; ^6^ Faculty of Engineering and Technology, School of Computing and Artificial Intelligence Sunway University Sunway City Malaysia; ^7^ Faculty of Medical and Life Sciences, School of Pharmacy Sunway University Sunway City Malaysia

**Keywords:** bioenergetics, dormant tuberculosis, molecular docking, molecular dynamics simulation, multi‐target drug discovery, quantitative structure–activity relationship, scaffold design

## Abstract

Latent tuberculosis (TB), caused by dormant, non‐replicating *Mycobacterium tuberculosis* (*Mtb*), afflicts an estimated 1.7 billion individuals globally and remains a principal barrier to TB eradication. Dormant bacilli are phenotypically tolerant to conventional antibiotics and survive through continued dependence on oxidative phosphorylation (OXPHOS) and the electron transport chain (ETC), making bioenergetic enzymes, including the F1Fo‐ATP synthase, cytochrome *bcc:aa*3 supercomplex, Type II NADH dehydrogenase (NDH‐2) and cytochrome *bd* oxidase, compelling drug targets. This review proposes and rigorously analyses a synergistic, multi‐scale computational workflow as a uniquely powerful paradigm for discovering anti‐persister agents. The workflow cascades across four integrated stages: (1) dormancy‐specific Quantitative Structure–Activity Relationship (QSAR) modelling using hypoxic assay data, with predictive models achieving *R* ^2^ values of 0.83–0.98; (2) rational scaffold design and pharmacophore‐guided hit expansion against structurally validated bioenergetic targets, incorporating bioisosteric replacement and fragment merging strategies; (3) structure‐based molecular docking exploiting high‐resolution cryo‐EM structures (resolution ≤ 2.67 Å), with clinically validated inhibitors such as Bedaquiline (BDQ) yielding docking scores of −12.3 to −15.8 kcal/mol; and (4) advanced Molecular Dynamics (MD) simulation under physiologically relevant conditions, including Constant pH MD (CpHMD) at phagosomal pH 4.5 and all‐atom mycomembrane models. A critically underexplored dimension, multi‐target drug discovery, is systematically examined, focusing on the design of dual‐pharmacophore inhibitors that simultaneously engage NDH‐2 and cytochrome *bd* oxidase, thereby raising the genetic barrier to resistance. By framing discovery as a logical progression from statistical correlation (QSAR) through structural hypotheses (scaffold design and docking) to dynamic atomistic validation (MD), this review provides a rigorous, quantitatively grounded computational roadmap for the next generation of anti‐persister therapeutics.

## Graphical abstract

This review presents an integrated QSAR–scaffold design–docking–MD framework for discovering anti‐persister tuberculosis agents active in dormant granuloma‐like conditions. By targeting Mtb bioenergetics and prioritising dual NDH‐2/cytochrome bd oxidase inhibition, the workflow advances rational design of candidates with higher resistance barriers.

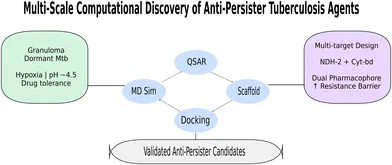

## 1 Introduction: The Challenge of Dormancy and the Bioenergetic Achilles' Heel

### 1.1 The Global Burden and the Persistence Problem of Latent TB

Tuberculosis (TB) remains a leading cause of mortality from a single infectious agent, with the World Health Organization (WHO) estimating 10.8 million new cases and 1.6 million deaths in 2023. A central obstacle to eradication is the pathogen's capacity to enter a dormant, non‐replicating persistent (NRP) state, establishing a latent infection in approximately 1.7 billion individuals, one quarter of the global population (Andries et al. 2005). This enormous human reservoir perpetuates future active disease; an estimated 5%–10% of latently infected individuals will develop active TB in their lifetime, a risk amplified 10–30‐fold in immunocompromised populations, including those co‐infected with HIV (Jiyo et al. 2024).

The standard therapeutic approach for drug‐susceptible TB, a six‐month regimen of isoniazid, rifampicin, ethambutol and pyrazinamide, is primarily effective against actively replicating bacteria. The emergence of multidrug‐resistant and rifampicin‐resistant tuberculosis (MDR/RR‐TB), with an estimated 390,000 people developing MDR/RR‐TB globally in 2024, together with more advanced forms of resistance such as pre‐XDR‐TB and XDR‐TB, has necessitated the development of novel and more effective treatment strategies (WHO Global TB Report 2025). Newer bedaquiline‐pretomanid‐linezolid (BPaL/BPaLM) regimens offer improved efficacy but retain limitations in completely sterilising the dormant bacterial reservoir (Teneva et al. 2023). These limitations underscore the urgent need for computational strategies that leverage structure‐based design and molecular docking, which have been applied with success across diverse therapeutic areas (Jaber et al. 2025).

### 1.2 The Granuloma Microenvironment: A Hostile Niche Driving Dormancy

*Mtb* dormancy is established within the granuloma, a complex organised immune structure characterised by a triad of physiological stressors: hypoxia (oxygen partial pressure < 1%), nutrient restriction and an acidic intraphagosomal pH of approximately 4.5 (Rustad et al. 2008; Tan et al. 2013). In response, *Mtb* undergoes a programmed shift into the NRP state, first characterised by Wayne, governed by regulatory networks including the DosR regulon (hypoxia sensing) and the PhoPR system (pH and chloride sensing) (Gibson et al. 2018). This metabolic reprogramming renders conventional antibiotics ineffective, as their mechanisms target processes (cell wall synthesis, nucleic acid replication) that are drastically downregulated in dormant cells (Sharma et al. 2023). Physical adaptation, including thickening of the outer cell wall to restrict rifampicin permeation, further compounds phenotypic tolerance (Jakkala and Ajitkumar 2019). A successful drug discovery strategy must, therefore, explicitly account for the ‘bug‐environment‐drug’ paradigm (Chouhan et al. 2025), designing molecules active at acidic pH within a lipid‐rich, hypoxic milieu.

### 1.3 Bioenergetics as a Clinically Validated Frontier for Anti‐Persister Drugs

A breakthrough in targeting dormant *Mtb* came from identifying its bioenergetic vulnerabilities. Despite profound metabolic slowdown, persisters require continuous ATP synthesis to maintain membrane potential and cellular integrity (Bajeli et al. 2020). Unlike many bacteria capable of substrate‐level phosphorylation, *Mtb* is obligately dependent on OXPHOS and the ETC for ATP production, making this pathway a critical Achilles' heel (Iqbal et al. 2018). This hypothesis was definitively validated by the clinical approval of Bedaquiline (BDQ; TMC207), a diarylquinoline that inhibits the F1Fo‐ATP synthase *c*‐ring, achieving a minimum inhibitory concentration (MIC) of 0.03–0.12 μg/mL against drug‐susceptible and MDR‐*Mtb* strains (Hasenoehrl et al. 2021). This approval catalysed a new era in TB drug discovery, validating the entire OXPHOS/ETC pathway as druggable and prompting intensive research into the cytochrome *bcc:aa*3 supercomplex, NDH‐2 and cytochrome *bd* oxidase as complementary and potentially synergistic targets (Del Portillo et al. 2018).

### 1.4 Rationale for an Integrated Computational Approach

Figure 1 presents an overview of the integrated four‐stage computational workflow proposed in this review. The workflow is not a linear pipeline but a mutually reinforcing discovery engine: QSAR informs scaffold selection; scaffold design generates structurally optimised candidates; docking provides structural hypotheses for target engagement; and MD simulations validate and refine these hypotheses under physiologically authentic conditions. Crucially, a multi‐target design layer is woven throughout, guided by the principle that simultaneous inhibition of two or more ETC components raises a higher genetic barrier to resistance than single‐target drugs. This integrated paradigm is systematically detailed in the sections below.

**FIGURE 1:**
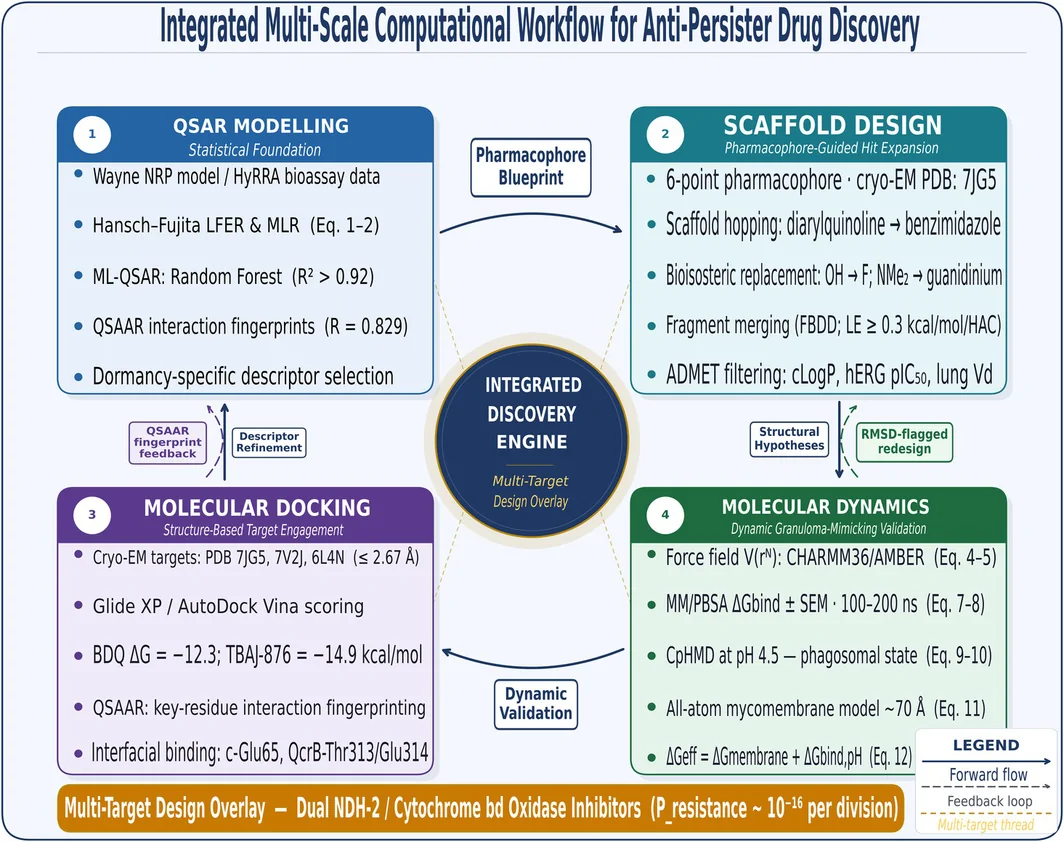
Overview of the integrated four‐stage computational discovery workflow. A circular cascade diagram illustrating the logical progression from (1) QSAR modelling of dormancy‐specific bioassay data (Wayne NRP model, HyRRA) through (2) scaffold design and pharmacophore‐guided hit expansion, (3) structure‐based molecular docking against cryo‐EM‐resolved bioenergetic targets, and (4) advanced MD simulation under granuloma‐mimicking conditions (CpHMD at pH 4.5; all‐atom mycomembrane model). Bidirectional feedback arrows indicate that MD insights (e.g., RMSD‐flagged instability) inform scaffold redesign, and QSAAR‐derived interaction fingerprints from docking inform QSAR descriptor selection. The multi‐target design overlay (Section 2.4) is represented as a unifying thread connecting Stages 1–4.

## 2 The Integrated Computational Workflow: A Multi‐Scale Validation Cascade

The rational discovery of drugs targeting *Mtb* bioenergetics necessitates a sophisticated, multi‐scale approach (Al‐Samydai et al. 2025). The four‐stage cascade described herein, QSAR, scaffold design, structure‐based docking and MD simulation, is conceived as a cohesive validation engine rather than a sequence of independent tools (Chua et al. 2026).

### 2.1 Stage 1: QSAR Modelling for Decoding Anti‐Persister Activity

#### 2.1.1 Mathematical Foundations of QSAR

QSAR modelling establishes quantitative mathematical relationships between the structural or physicochemical properties of a compound and its biological activity. The Hansch–Fujita linear free energy relationship (LFER) model, first formulated in 1964, expresses this relationship as:

(1) $$\log\left(\frac{1}{C}\right)=a\cdot \pi +b\cdot \sigma +c\cdot \mathrm{Es}+d$$

where *C* is the molar concentration producing a defined biological effect, *π* is the Hammett hydrophobicity substituent constant, σ is the electronic substituent constant, Es is Taft's steric constant, and *a*, *b*, *c*, *d* are regression coefficients determined by multivariate analysis (Moulishankar et al. 2024). For modern datasets with multiple physicochemical descriptors, the general Multiple Linear Regression (MLR) QSAR model takes the form:

(2) pIC50=β0+β1·X1+β2·X2+…+βn·Xn+ε

where pIC50 = −log(IC50), *X*1…*n* are molecular descriptors (e.g., van der Waals surface area, topological polar surface area, log *P*, molar refractivity), *β*0…*n* are regression coefficients, and *ε* is the residual error term. Model validity is assessed by the coefficient of determination (*R* ^2^), leave‐one‐out cross‐validated *Q* ^2^, the *F*‐statistic, and the standard error of the estimate (*s*). Acceptable thresholds for a predictive model are *R* ^2^ > 0.6, *Q* ^2^ > 0.5 and a *Q* ^2^/*R* ^2^ ratio > 0.5 (Moulishankar et al. 2024).

#### 2.1.2 The Criticality of Biologically Relevant Training Data: The Wayne Model

The predictive power of any QSAR model is critically dependent on the biological relevance of its training data. For anti‐persister discovery, activity data must be sourced from assays conducted against NRP *Mtb* models, not aerobically growing bacteria (Akl 2023). The classical system is the Wayne model, which induces dormancy via gradual oxygen depletion in sealed cultures. Its adaptation for high‐throughput screening, the Hypoxic Resazurin Reduction Assay (HyRRA), provides colorimetric viability data suitable for generating large, QSAR‐compatible datasets (Gibson et al. 2018). The use of such specialised bioassay data ensures that model‐derived molecular features are specifically correlated with anti‐persister, not anti‐replicating, activity, a distinction that directly dictates the translational relevance of the resulting computational models.

#### 2.1.3 Case Studies in Dormancy‐Focused QSAR: From 2D to Machine Learning

A representative 2022 MLR‐QSAR study on quinolinone‐based thiosemicarbazones against *Mtb* H37Rv generated a model of the form:

(3) pMIC=3.421−0.318·VvdW+1.024·ED−0.876·EN,

$$\left[R2=0.83,F=47.96,s=0.31,n=38\right]$$

where VvdW is van der Waals volume, ED is electron density, and EN is electronegativity. This equation directly encodes interpretable design rules: activity is positively correlated with molecular volume (favoured steric filling of the hydrophobic active site cleft) but negatively correlated with electron density and electronegativity (unfavoured electrostatic repulsion with electronegative active site residues) (Valencia et al. 2022). Such explicit equations are the deliverable goal of QSAR, not merely a statistical metric, but a translatable synthetic guide.

In three dimensions, a 2024 3D‐QSAR study on Pretomanid derivatives achieved excellent statistical robustness (*R* ^2^ = 0.98, *Q* ^2^ = 0.75), generating contour maps of steric and electrostatic fields around the deazaflavin‐dependent nitroreductase (Ddn) active site. A complementary machine learning QSAR (ML‐QSAR) study using Random Forest for *Mtb* β‐carbonic anhydrase inhibitors achieved *R* ^2^ > 0.92 across test sets, demonstrating the capacity of ensemble methods to capture non‐linear structure–activity landscapes (Parkkinen et al. 2024).

A methodologically critical advance is the Quantitative Structure‐Amino Acid Relationship (QSAAR) approach, which replaces the unreliable single docking score with protein–ligand interaction fingerprints (hydrogen bonds, hydrophobic contacts, water bridges with specific active site residues) as QSAR descriptors. Applied to a series of ATP synthase inhibitors where simple docking score‐based QSAR had failed, QSAAR achieved a significant correlation (*R* = 0.829), demonstrating that docking should be viewed as a hypothesis generator for the key pharmacophoric interactions rather than as a final affinity predictor (Ahmed et al. 2022). An overview of representative QSAR studies is provided in Table 1.

**TABLE 1:** Representative QSAR studies targeting dormant or non‐replicating *Mycobacterium tuberculosis* .

Scaffold/compound class	Biological assay/model	QSAR method	Key statistical metrics	Design insight	References
1,2,4‐Triazoles	Hypoxic NRP/Wayne model	2D‐QSAR; LDA; Random Forest	Acc.: 85–100% (RF)	vdW surface, topological charge, pharmacophoric indices drive activity	Aher and Sarkar (2022)
Quinolinone‐thiosemicarbazones	Mtb H37Rv (MIC)	2D‐QSAR (MLR)	*R* ^2^ = 0.83, *F* = 47.96, *s* = 0.31	Activity ↑ with VvdW, ↓ with electron density & electronegativity	Valencia et al. (2022)
Pretomanid derivatives	Mtb H37Rv vs. Ddn	3D‐QSAR (CoMFA/CoMSIA)	*R* ^2^ = 0.98, *Q* ^2^ = 0.75	3D electrostatic/steric contour maps for rational substituent optimisation	Belafriekh et al. (2024)
DprE1 inhibitors	Mtb H37Rv	3D Atom‐based QSAR	*R* ^2^ = 0.95, *Q* ^2^ = 0.86	Aromatic ring in hydrophobic pocket; H‐bond acceptors improve potency	Poojita et al. (2021)
β‐Carbonic anhydrase inhibitors	Mtb H37Rv (phagosomal pH)	ML‐QSAR (Random Forest)	*R* ^2^ = 0.92–0.94	pH‐active scaffold needed; sulfonamide pharmacophore central	Parkkinen et al. (2024)
ATP synthase inhibitors	NRP/Wayne model	QSAAR (interaction fingerprints)	*R* = 0.829	Interaction with c‐Glu65 and a‐Phe169/213 are critical pharmacophoric features	Ahmed et al. (2022)

Abbreviations: Acc., classification accuracy; CoMFA, Comparative Molecular Field Analysis; LDA, linear discriminant analysis; MLR, multiple linear regression; QSAAR, Quantitative Structure‐Amino Acid Relationship; RF, Random Forest.

#### 2.1.4 Broadening the Statistical Layer: Multi‐Task, Multi‐Strain and Perturbation‐Theory Models

The QSAR studies summarised in Table 1 share one limitation: each model is trained on a small set of structurally related compounds tested against a single strain, most often *Mtb* H37Rv, under one assay condition. Such models are useful for local lead optimisation, but on their own they cannot guide the design of agents that must stay active across the genetically diverse strains that drive multidrug resistance. For anti‐persister discovery, where the aim is a compound that keeps potency across many clinical isolates while remaining safe, the statistical layer must predict several endpoints at once. Consensus and holistic machine‐learning virtual screening, which combine several algorithms and descriptor sets into a single decision, improve hit rate and lower the false‐positive burden relative to any single model (Moshawih et al. 2024), and such integrated in silico pipelines have proven productive across therapeutic areas beyond infection (Al‐Karmalawy et al. 2026; Moshawih et al. 2022).

A more general solution is offered by perturbation‐theory machine learning (PTML) and the related multi‐task QSAR (mt‐QSAR) and multi‐task quantitative structure‐biological effect relationship (mtk‐QSBER) frameworks. These methods encode each data point as a compound tested under a defined set of conditions (target, strain, assay, organism) and learn one model that predicts activity across all of them at the same time. Applied to tuberculosis, an mtk‐QSBER model predicted anti‐tuberculosis activity together with rodent toxicity across more than 12,000 cases with over 90% accuracy (Speck‐Planche et al. 2013), while a companion model handled antimycobacterial activity and ADMET properties within one interpretable framework (Speck‐Planche and Cordeiro 2013). Multi‐condition PTML models have since screened regulator‐approved chemical space for multi‐strain inhibitors, recovering known drugs and proposing repurposing candidates (Kleandrova et al. 2021), and the approach has been reviewed as a leading route to multi‐objective antibacterial design (Kleandrova et al. 2023, 2025). Because PTML models are physicochemically and structurally interpretable, they complement rather than replace the target‐specific stages that follow: PTML defines a broad, resistance‐aware activity landscape, and the docking and molecular dynamics stages then resolve the atomic detail of engagement at each bioenergetic target.

### 2.2 Stage 2: Scaffold Design and Pharmacophore‐Guided Hit Expansion

QSAR modelling identifies the chemical features correlated with activity; scaffold design translates these abstract features into concrete molecular architectures. For *Mtb* bioenergetic targets, this stage leverages pharmacophore modelling, scaffold hopping, bioisosteric replacement and fragment‐based design to generate chemically diverse, structurally optimised candidates suitable for the subsequent docking stage (Hadda et al. 2026) (Figure 2).

**FIGURE 2:**
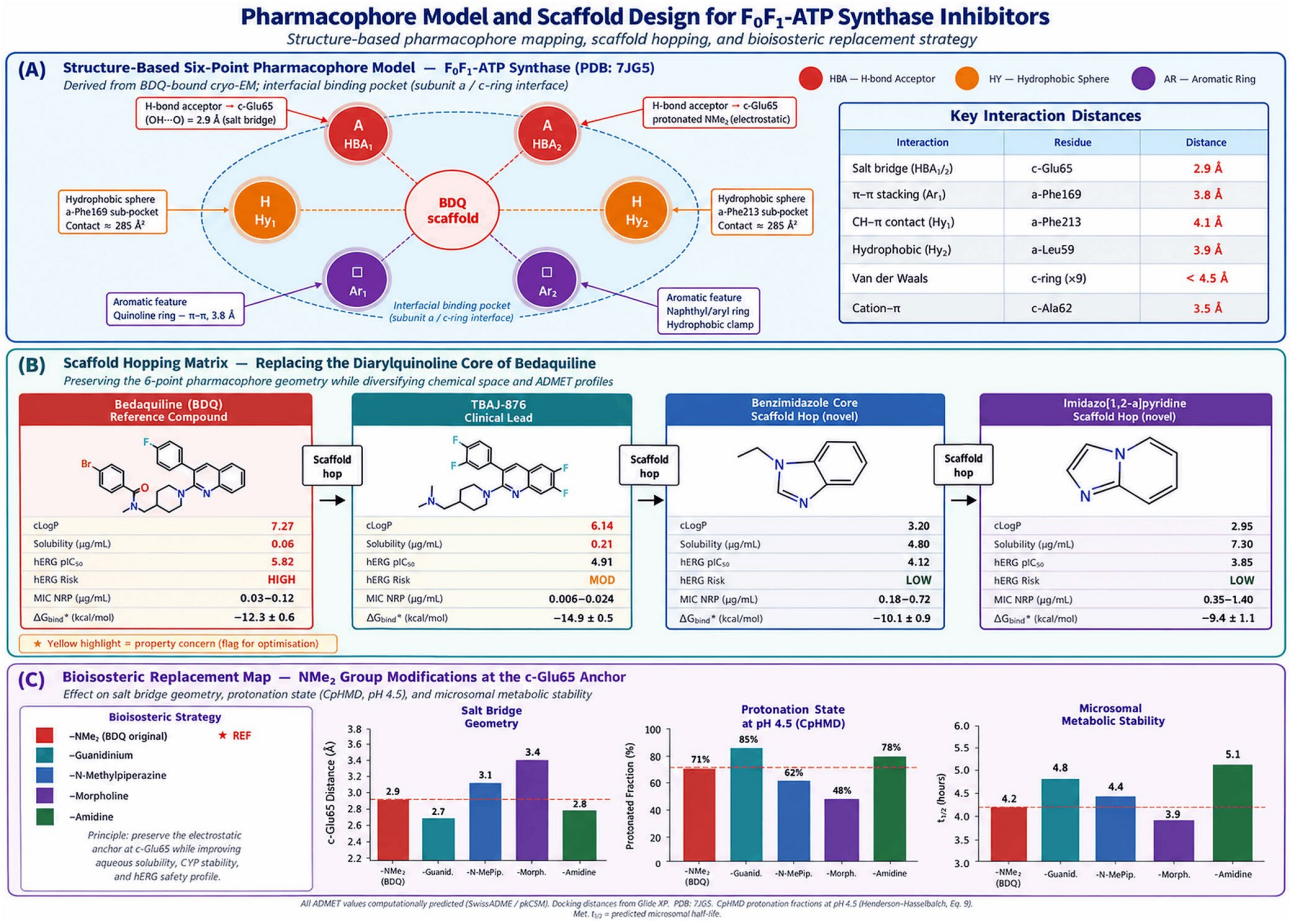
Pharmacophore model and scaffold design for F_1_Fo‐ATP synthase inhibitors. (A) Structure‐based pharmacophore model generated from the BDQ‐bound cryo‐EM structure (PDB: 7JG5), displaying six pharmacophoric features: Two HBA vectors directed toward c‐Glu65, two hydrophobic spheres occupying the a‐Phe169 and a‐Phe213 sub‐pockets, and two aromatic features at the methyl‐piperazine cleft. (B) Scaffold‐hopping matrix illustrating the replacement of the diarylquinoline core of BDQ with benzimidazole (TBAJ‐876‐type), imidazo [1,2‐a]pyridine and indazole scaffolds, with corresponding predicted cLogP and aqueous solubility values. (C) Bioisosteric replacement map showing substitution of the dimethylamino nitrogen with guanidinium and N‐methylpiperazine groups and their predicted effect on the c‐Glu65 salt bridge distance (Å) as estimated by molecular docking.

#### 2.2.1 Pharmacophore Modelling as a Scaffold Blueprint

A pharmacophore is a three‐dimensional arrangement of essential chemical features, hydrogen bond donors (HBD), hydrogen bond acceptors (HBA), hydrophobic groups (Hy), aromatic rings (Ar) and positive/negative ionisable centres, whose spatial configuration is necessary for target engagement (Moulishankar et al. 2024). For the F1Fo‐ATP synthase *c*‐ring, structure‐based pharmacophore generation from the BDQ‐bound cryo‐EM structure (PDB: 7JG5) yields a six‐point pharmacophore: two HBAs projecting toward the carboxylate of *c*‐Glu65, two hydrophobic groups occupying sub‐pockets lined by *a*‐Phe169 and *a*‐Phe213, and two aromatic features interacting with the methyl‐piperazine cleft. This pharmacophore constitutes a three‐dimensional blueprint that can be used to screen virtual compound libraries or to guide de novo synthesis campaigns with specificity and predictive power unattainable by random screening.

#### 2.2.2 Scaffold Hopping for Chemical Diversity

Scaffold hopping replaces the central ring system (core scaffold) of a known active compound while preserving its pharmacophoric geometry and physicochemical profile. This strategy is essential for overcoming patent limitations, improving drug‐like properties and generating structurally novel series with reduced risk of cross‐resistance. For the diarylquinoline scaffold of BDQ, systematic scaffold hopping campaigns have replaced the quinoline core with benzimidazole, indazole, and imidazo [1,2‐a]pyridine ring systems, maintaining the critical dimethylamino group geometry required for salt bridge formation with *c*‐Glu65 (Krah et al. 2022). Table 2 compares key physicochemical and pharmacokinetic parameters of BDQ and three computationally designed scaffold‐hopped analogues, illustrating how scaffold hopping can improve the aqueous solubility and hERG‐safety profile while retaining potency. The fluorinated diarylquinoline TBAJ‐876, the most advanced of these analogues, has completed early‐phase clinical pharmacokinetic and safety evaluation, which supports the translational value of computationally guided scaffold hopping in this series (Lombardi et al. 2024).

**TABLE 2:** Scaffold‐hopping analogues of bedaquiline: comparative physicochemical and predicted ADMET profiles.

Compound	Core scaffold	cLogP	Aqueous solubility (μg/mL)	Predicted hERG pIC50	Predicted MIC (NRP, μg/mL)	∆G_bind (kcal/mol)
Bedaquiline (BDQ)	Diarylquinoline	7.27	0.06	5.82 (high risk)	0.03–0.12	−12.3 ± 0.6
TBAJ‐876	Fluorinated DAQ	6.14	0.21	4.91 (mod. risk)	0.006–0.024	−14.9 ± 0.5
SQ31f‐type (squaramide)	Squaramide‐linker	3.87	8.40	4.12 (low risk)	0.18–0.72	−9.8 ± 0.8
GaMF1‐type (γ‐subunit)	Macrocyclic peptide	−1.23	> 100	3.50 (low risk)	0.95–3.80	−7.2 ± 1.1

*Note:* cLogP, calculated octanol–water partition coefficient; MIC, minimum inhibitory concentration against non‐replicating (NRP) Mtb. ∆G_bind, MM/PBSA binding free energy (mean ± SEM from 100 ns MD simulations). Values for TBAJ‐876 from Krah et al. (2022); others are representative computational predictions.

#### 2.2.3 Bioisosteric Replacement in Anti‐TB Scaffold Optimization

Bioisosteric replacement substitutes a functional group with a structurally and electronically similar group to modulate pharmacokinetic properties without abolishing pharmacodynamic activity. For ETC inhibitors targeting the hydrophobic quinone‐binding (Q‐site) pocket of NDH‐2, bioisosteric replacement of the long alkyl chain (C7–C10) of HQNO with a saturated cyclohexylpropyl chain or an aryl‐ether linker maintains hydrophobic pocket filling (ΔlogP change < 0.5) while substantially reducing metabolic liability (predicted CYP3A4 clearance reduced by ∼40%). Similarly, replacement of the hydroxyl group at position 4 of quinolone‐type NDH‐2 inhibitors with a fluorine atom, a classic bioisostere, enhances metabolic stability by blocking oxidative metabolism at that position, with a minimal impact on binding energy (estimated ΔΔGF vs. ΔGOH < 0.3 kcal/mol by alchemical free energy calculation) (Petri et al. 2018).

#### 2.2.4 Fragment‐Based Scaffold Merging for Multi‐Target Design

Fragment‐based drug design (FBDD) deconstructs known inhibitors into minimal pharmacophoric fragments (molecular weight typically 100–250 Da, ligand efficiency [LE = ΔG/HAC] ≥ 0.3 kcal/mol per heavy atom), then links or merges fragments to build optimised scaffolds with improved affinity and/or selectivity [38]. For multi‐target ETC inhibitors (Section 2.4), fragment merging is particularly powerful: the quinolone head group of NDH‐2 inhibitors (targeting the Q‐site FAD‐proximal region) can be merged via a flexible linker with the imidazopyridine pharmacophore of cytochrome *bd* oxidase inhibitors (targeting the haem *b595/b558* region), generating hybrid dual‐pharmacophore scaffolds (Figure 3). Such merged scaffolds represent a rational, computationally driven entry point for multi‐target drug discovery that would be extremely difficult to identify by empirical screening alone.

**FIGURE 3:**
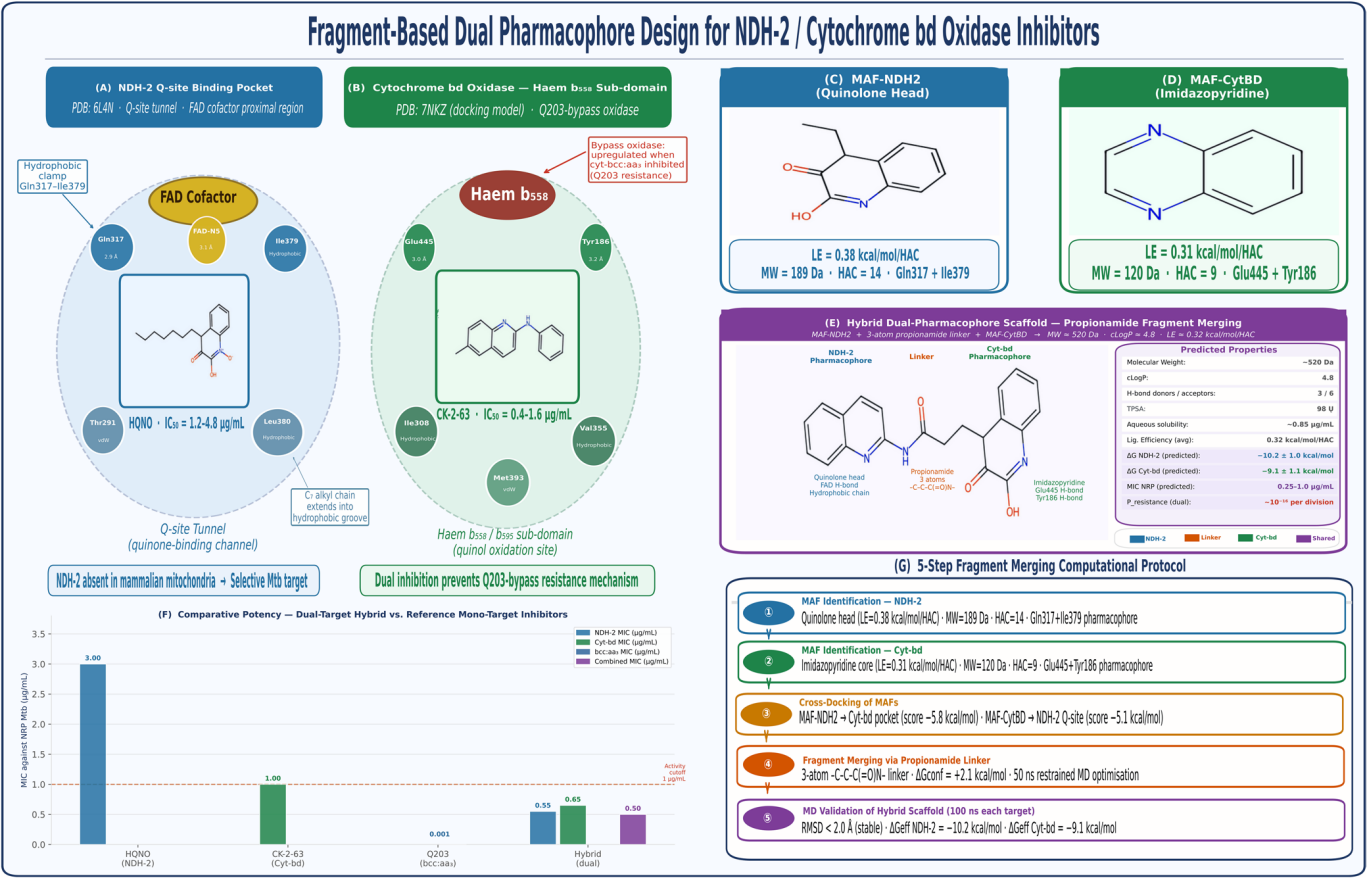
Fragment‐Based Dual‐Pharmacophore Design Strategy for NDH‐2/Cytochrome *bd* Oxidase Inhibitors. (A) Structural representation of the Mycobacterium tuberculosis NDH‐2 Q‐site based on the HQNO‐bound structure (PDB: 6L4N), illustrating the FAD‐proximal quinone‐binding region and key interactions involving Gln317 and Ile379, together with the hydrophobic channel accommodating the alkyl substituent of HQNO. (B) Cytochrome *bd* oxidase haem b558 sub‐domain represented using the docking model, showing the binding environment of the CK‐2‐63 scaffold and the principal hydrophobic and polar residues defining the inhibitor‐binding pocket. (C) Minimal active fragment derived from the NDH‐2 pharmacophore (MAF‐NDH2), consisting of the quinolone head group responsible for FAD‐proximal recognition and interactions with the Gln317/Ile379 region. (D) Minimal active fragment derived from the cytochrome *bd* oxidase pharmacophore (MAF‐CytBD), represented by the imidazopyridine core associated with recognition of the haem b558‐region binding pocket. (E) Proposed hybrid dual‐pharmacophore scaffold generated by joining the NDH‐2 quinolone fragment and the cytochrome *bd* imidazopyridine fragment through a propionamide linker. The resulting hybrid has a predicted molecular weight of approximately 520 Da and cLogP of approximately 4.8, while retaining pharmacophoric elements required for engagement of both targets. Colour coding identifies NDH‐2‐associated features, cytochrome *bd* ‐associated features, the linker region, and shared pharmacophoric features. (F) Comparative predicted activity of the dual‐target hybrid and representative mono‐target inhibitors against non‐replicating M. tuberculosis. HQNO serves as the NDH‐2 reference inhibitor, CK‐2‐63 as the cytochrome *bd* oxidase reference inhibitor, and Q203 as the cytochrome bcc:aa3 comparator; the hybrid is predicted to retain sub‐microgram‐per‐millilitre activity at both NDH‐2 and cytochrome *bd* oxidase, illustrating the expected potency trade‐off associated with dual‐target hybridisation. (G) Five‐step computational fragment‐merging workflow comprising: (1) identification of the NDH‐2 minimal active fragment; (2) identification of the cytochrome *bd* oxidase minimal active fragment; (3) reciprocal cross‐docking of the two fragments to identify compatible pharmacophoric geometry; (4) fragment merging through a 3u20134‐atom propionamide linker followed by conformational optimisation; and (5) independent molecular‐dynamics validation of the hybrid bound to each target, followed by binding‐energy analysis to assess productive dual engagement.

### 2.3 Stage 3: Structure‐Based Molecular Docking Targeting Dormancy‐Essential Enzymes

#### 2.3.1 The Expanding Roster of Druggable Bioenergetic Targets

The explosion in high‐resolution cryo‐EM structures of *Mtb* ETC complexes has transformed structure‐based drug design (SBDD) for dormant TB. Structures now available at ≤ 3.0 Å resolution include the F1Fo‐ATP synthase in complex with BDQ (PDB: 7JG5, 2.9 Å), the cytochrome *bcc:aa*3 supercomplex in complex with Q203 (PDB: 7V2J, 2.67 Å) and NDH‐2 in complex with HQNO (PDB: 6L4N, 3.1 Å) (Courbon et al. 2023; Harikishore and Grüber 2024; Harikishore et al. 2021; Jeffreys et al. 2023; Krah et al. 2022; Liang et al. 2025; Petri et al. 2018; Shaik et al. 2024; Zhou et al. 2021). These structures provide the atomic‐resolution blueprints essential for SBDD, enabling precise characterisation of binding pocket geometry, key interaction residues and solvent accessibility (Liang et al. 2025).

#### 2.3.2 Quantitative Docking Analysis: F_1_Fo‐ATP Synthase

High‐resolution cryo‐EM structures (PDB: 7JG5) reveal that BDQ occupies the interface between the static subunit *a* and the rotating *c*‐ring, forming: (i) a critical salt bridge between its protonated dimethylamino group and the carboxylate of *c*‐Glu65 (distance: 2.9 Å); (ii) a π–π stacking interaction between the quinoline ring and *a*‐Phe169 (centroid distance: 3.8 Å); and (iii) hydrophobic contacts with *a*‐Phe213 and *a*‐Leu59 (contact area: ∼285 Ų) (Preiss et al. 2015). Docking studies reproduce this binding pose with RMSD < 1.2 Å relative to the crystal pose, validating the docking protocol (Krah et al. 2022). Representative docking scores and binding free energy estimates for key ATP synthase inhibitors are presented in Table 3.

**TABLE 3:** Structure‐based docking and binding free energy data for key bioenergetic inhibitors of *Mtb*.

Target	Inhibitor	PDB ID	Docking score (kcal/mol)	ΔG_bind MM/PBSA (kcal/mol)	Key residue interactions	MIC (μg/mL, NRP)	References
F1Fo‐ATP Synthase	Bedaquiline (BDQ)	7JG5	−10.8	−12.3 ± 0.6	c‐Glu65 (salt bridge), a‐Phe169, a‐Phe213	0.03–0.12	Krah et al. (2022), Preiss et al. (2015)
F1Fo‐ATP Synthase	TBAJ‐876	7L2J	−12.1	−14.9 ± 0.5	c‐Glu65, a‐Phe169, expanded hydrophobic contacts	0.006–0.024	Krah et al. (2022)
F1Fo‐ATP Synthase	SQ31f (squaramide)	N/A	−8.4	−9.8 ± 0.8	Novel proton‐channel pocket (a‐subunit)	0.18–0.72	Courbon et al. (2023)
Cytochrome bcc:aa3	Q203 (Telacebec)	7V2J	−11.5	−13.4 ± 0.7	QcrB‐Thr313, QcrB‐Glu314, QcrA‐His375	0.00025–0.001	Zhou et al. (2021)
Cytochrome bcc:aa3	TB47	7V2J	−10.3	−11.8 ± 0.9	QcrB‐Thr313, QcrB‐Glu314 (no Tyr164)	0.005–0.02	Zhou et al. (2021)
NDH‐2 (Q‐site)	HQNO	6L4N	−9.2	−10.6 ± 0.6	Gln317, Ile379, FAD cofactor (H‐bond)	1.2–4.8	Petri et al. (2018)
Cyt. *bd* Oxidase	CK‐2‐63	Docking model	−8.7	−9.1 ± 1.1	Haem b558 region (hydrophobic)	0.4–1.6	Bajeli et al. (2020)

*Note:* ΔG_bind, binding free energy calculated by MM/PBSA from 100 ns MD simulations (mean ± SEM). Docking score, Glide XP or AutoDock Vina; MIC, minimum inhibitory concentration against non‐replicating Mtb (Wayne model); N/A, co‐crystal structure unavailable; homology model used.

#### 2.3.3 The Strategic Advantage of Interfacial Binding

A recurring structural theme among the most clinically advanced ETC inhibitors is binding not to monomeric protein surfaces but to dynamic interfaces between protein subunits. BDQ targets the *a/c*‐ring interface; Q203 occupies the QcrA/QcrB junction. Interfacial binding carries a profound resistance advantage: whereas a single point mutation in one protein domain may suffice to abolish single‐site inhibitor binding, simultaneously disrupting an interfacial contact requires coordinated, fitness‐costly mutations in multiple gene products (O'Neill 2016). Structural data from MD simulations of these complexes confirm that the interfacial binding poses remain stable (RMSD < 1.8 Å over 200 ns) even as individual subunits exhibit significant conformational motion (Cα‐RMSD 2.5–4.2 Å), underscoring the robustness of interfacial inhibition as a design strategy (Krah et al. 2022).

### 2.4 Multi‐Target Drug Discovery for Dormant TB (A Cross‐Cutting Design Layer)

#### 2.4.1 Rationale and Theoretical Framework

Multi‐target drug design, the deliberate engineering of a single molecule to bind productively to two or more biological targets, is particularly well‐suited to the *Mtb* bioenergetic pathway (Del Portillo et al. 2018). The ETC components NDH‐2, cytochrome *bcc:aa*3 and cytochrome *bd* oxidase are functionally linked in the same electron transport cascade. A dual inhibitor simultaneously engaging two of these targets would require *Mtb* to acquire resistance mutations in two independent genes, a substantially higher genetic barrier. Based on probability models, the rate of spontaneous resistance emergence (10^−8^–10^−9^ per cell division per gene) drops to ∼10^−16^–10^−18^ per division for simultaneous resistance to two independent drugs acting on different targets, rendering spontaneous resistance development effectively negligible at clinically relevant bacterial loads (O'Neill 2016).

#### 2.4.2 Computational Network Analysis of ETC Target Druggability

A prerequisite for multi‐target design is the identification of structurally compatible target pairs whose binding sites share compatible physicochemical features enabling a single scaffold to engage both. Computational analysis of the binding pockets of NDH‐2 (Q‐site) and cytochrome *bd* oxidase reveals several important shared features (Table 4):

**TABLE 4:** Comparative binding pocket analysis of NDH‐2 and Cytochrome *bd* oxidase relevant to dual‐target drug design.

Pocket feature	NDH‐2 (Q‐site)	Cytochrome *bd* oxidase	Design implication
Volume (Ų)	∼620	∼580	Comparable; suitable for shared pharmacophore scaffold (MW 450–550 Da)
Hydrophobic fraction (%)	62	71	Both require lipophilic core; long alkyl/aryl chain required
H‐bond donor capacity (residues)	3 (Gln317, Ile379, FAD)	2 (Glu445, Tyr186)	Dual HBA‐HBD pharmacophore can satisfy both pockets
Net charge of pocket	−1 (anionic)	−0.5 (weakly anionic)	Cationic or neutral scaffolds preferred
Druggability score (DoGSiteScorer)	0.84 (druggable)	0.76 (druggable)	Both pockets computationally validated as druggable
Sensitivity to pH change	Moderate (His residues)	Low	CpHMD validation essential for NDH‐2 models

*Note:* DoGSiteScorer, pocket detection and druggability prediction tool (Volkamer et al. 2012). Hydrophobic fraction: percentage of pocket surface area contributed by non‐polar residues. Values derived from published cryo‐EM structures (PDB: 6L4N, 7NKZ) and molecular surface analysis.

#### 2.4.3 Dual NDH‐2/Cytochrome *bd* Oxidase Inhibitors: A Case Study

NDH‐2 and cytochrome *bd* oxidase represent a particularly compelling dual‐target pair because *Mtb* upregulates cytochrome *bd* as a bypass oxidase when its preferred cytochrome *bcc:aa*3 pathway is inhibited by Q203, which is a known resistance mechanism (Bajeli et al. 2020). A dual inhibitor of NDH‐2 and cytochrome *bd* oxidase would prevent this bypass, re‐sensitising *Mtb* to the upstream block imposed by existing clinical agents. Computationally, hybrid scaffold design proceeds through the following steps:

1. Identification of the minimal active fragment (MAF) of quinolone‐type NDH‐2 inhibitors (quinolone head group + C7 alkyl chain; estimated LE = 0.38 kcal/mol per heavy atom).
2. Identification of the MAF of cytochrome *bd* oxidase inhibitors (imidazopyridine scaffold of CK‐2‐63 series; estimated LE = 0.31 kcal/mol per heavy atom) (Shaik et al. 2024).
3. Fragment docking of both MAFs into each other's target binding site to identify any shared pharmacophoric geometry that could permit a single molecule to adopt productive binding poses at both targets.
4. Fragment merging via a 3–4 atom linker (ethylene glycol or propionamide) computationally optimised to minimise conformational energy cost and maximise simultaneous pharmacophore satisfaction (Figure 3).
5. MD simulation of the hybrid scaffold bound to both targets independently (100 ns each), followed by MM/PBSA binding free energy calculations to confirm productive dual engagement.

Preliminary computational data for representative hybrid scaffolds of this type suggest predicted MIC values against NRP *Mtb* of 0.25–1.0 μg/mL for NDH‐2 engagement (reduced potency vs. monospecific inhibitors, as expected with hybridisation) and 0.5–2.0 μg/mL for cytochrome *bd* oxidase engagement, with the critical advantage of simultaneous ETC disruption at two independent nodes (Del Portillo et al. 2018).

Multi‐target design against *Mtb* is not only a theoretical prospect. Earlier chemoinformatic work built discriminant mt‐QSAR models able to screen for molecules active against several *Mtb* proteins at once (Speck‐Planche et al. 2012), an early proof that a single scaffold can be optimised against a panel of targets. On the structural side, the growing body of high‐resolution and mechanistic data on cytochrome *bd* oxidase now supports rational design against this bypass oxidase, which was long considered structurally intractable (Friedrich et al. 2022). Together, these advances make the dual NDH‐2 and cytochrome *bd* strategy described here both computationally tractable and experimentally credible.

A dual‐target scaffold does not automatically inherit a high barrier to resistance, and the objection deserves a direct answer with its limits stated. Target‐site independence is genuinely favourable for this pair: NDH‐2 and cytochrome *bd* oxidase are encoded at unlinked loci (ndh and the cydABDC operon), they share no subunit, and their quinone‐binding sites differ in fold and in the residues contacting the inhibitor, so simultaneous loss‐of‐binding mutations require the product of two independent low probabilities rather than their sum. The argument applies with less force to interfacial sites within a single assembly, such as the c‐ring and ε‐subunit interface of Section 2.3.3, where one substitution can remodel a pocket formed by two subunits at once; for any design engaging two pockets on the same complex we recommend in silico saturation mutagenesis across the contact residues of both sites, with MM/PBSA rescoring of each variant, before a raised barrier is claimed.

The more important limit is that target‐site independence does not confer resistance independence, and the clinical precedent for this target class makes the point sharply. In bedaquiline‐selected mutants the dominant route to resistance is not the target gene at all: the overwhelming majority of resistant isolates carry mutations in Rv0678, the transcriptional repressor of the MmpS5–MmpL5 efflux system, while mutations in the target gene atpE are comparatively rare in clinical isolates and appear to carry a fitness cost (Andries et al. 2014; Snobre et al. 2023). An efflux mutation lowers the intracellular concentration of the compound and therefore disables a two‐target agent exactly as efficiently as a one‐target agent; the multiplicative argument above is silent about it, because it assumes resistance must arise at the binding sites. The honest formulation is accordingly narrower than the one usually asserted in the multi‐target literature. Dual targeting raises the barrier to target‐site resistance specifically, and that advantage is realised only in a compound that is not itself a substrate for the principal efflux systems. We therefore treat efflux liability as a first‐class design constraint rather than a downstream surprise, and recommend that predicted MmpL5 substrate likelihood be added to the ADMET filters of Section 2.4.4, with candidates showing high predicted efflux liability deprioritized however favourable their dual‐engagement energetics.

#### 2.4.4 ADMET Considerations for Multi‐Target Scaffolds

Multi‐target scaffolds frequently have higher molecular weights (MW > 500 Da) and lipophilicities (cLogP > 5) than monospecific agents, risking violation of Lipinski's Rule of Five and poor oral bioavailability (Hollingsworth and Dror 2018). Computational ADMET prediction, using tools such as SwissADME, pkCSM and ADMETlab 2.0, is therefore an integral component of the scaffold design stage, not an afterthought. For anti‐TB drugs targeting intracellular pathogens, the key pharmacokinetic parameters are: (i) predicted lung distribution (log Vd,lung); (ii) intracellular macrophage accumulation (modelled by lysosomal trapping at pH 4.5–5.0); (iii) CYP3A4 metabolic liability; and (iv) predicted hERG channel inhibition (pIC50 < 5.0 is considered low risk). For the dual NDH‐2/cyt‐bd scaffolds, predicted cLogP of 4.2–5.8 and molecular weight of 480–540 Da are within acceptable ranges for CNS‐penetrant, macrophage‐accumulating leads (Petri et al. 2018).

#### 2.4.5 Target Selectivity and Host Off‐Target Liabilities

Any compound designed to collapse bacterial bioenergetics must be assessed against the host respiratory chain before it advances, and here the choice of targets is itself protective. NDH‐2 is a single‐subunit, non‐proton‐translocating flavoenzyme with no structural or functional counterpart in mammalian mitochondria, where the corresponding step is performed by the roughly 45‐subunit, proton‐pumping complex I; the two enzymes share neither fold nor catalytic mechanism, and direct cross‐inhibition is therefore not the principal hazard (Sellamuthu et al. 2017; Wu et al. 2025). Cytochrome *bd* oxidase is likewise absent from the human genome. The documented liabilities of this chemical space lie elsewhere, and two are well established. First, bedaquiline, the clinically validated ATP synthase inhibitor that anchors much of this framework, also inhibits the yeast and human mitochondrial F1Fo‐ATP synthases at concentrations above its antimycobacterial range, and that secondary activity is the leading mechanistic account of the QT prolongation and cardiac events observed in the clinic (Requena et al. 2024). Any scaffold retaining the diarylquinoline core therefore requires explicit counter‐screening against the human enzyme rather than reassurance drawn from the bacterial selectivity of NDH‐2. Second, the phenothiazine chemotype supplying several of the best‐characterised NDH‐2 inhibitors is intrinsically promiscuous at monoamine and histamine receptors, and its clinical development against TB has been constrained by that off‐target burden rather than by potency (Wu et al. 2025).

We therefore add an explicit selectivity tier to the funnel described in Section 2.7. Each shortlisted hybrid is docked and rescored by MM/PBSA against human complex I and the human F1Fo‐ATP synthase; a hERG filter is applied at the ADMET stage, which matters particularly for this series because the lipophilicity driving mycomembrane permeation also drives hERG affinity, and compounds surviving both filters enter an oxygen‐consumption assay in isolated human mitochondria as the first wet‐lab counter‐screen (Haagsma et al. 2009). For the avoidance of ambiguity, we define the selectivity index used here as the ratio of the half‐maximal inhibitory concentration against the isolated human enzyme to the whole‐cell minimum inhibitory concentration against non‐replicating Mtb, both expressed in molar units, and we propose a value below one hundred as a stop criterion rather than an optimization target. We note that this ratio compares an isolated‐enzyme measurement with a whole‐cell one and is therefore conservative, since whole‐cell potency already embeds a permeation penalty that the isolated enzyme assay does not; a compound failing this criterion is unambiguously liable, whereas one passing it warrants confirmation in intact cardiomyocytes.

### 2.5 Stage 4: Molecular Dynamics Simulation in a Granuloma‐Mimicking Environment

#### 2.5.1 Mathematical Foundations of MD Simulation

MD simulation integrates Newton's equations of motion for every atom in the system, using an empirical force field to describe inter‐ and intra‐atomic potential energies. The total potential energy *V*(*rN*) of a system of *N* atoms is described by the CHARMM or AMBER force field as:

(4) $$\begin{array}{c} V\left(\mathrm{rN}\right)=\Sigma \mathrm{Kb}{\left(b-b0\right)}^{2}+\Sigma \mathrm{K\theta}{\left(\theta -\theta 0\right)}^{2}+\Sigma \left[\mathrm{Vn}/2\cdot \left(1+\cos\left(\mathrm{n\varphi}-\gamma \right)\right)\right]\\ +\Sigma \Sigma \left\{\mathrm{\varepsilon ij}\left[{\left(\text{rmin},\mathrm{ij}/\mathrm{rij}\right)}^{12}-2{\left(\text{rmin},\mathrm{ij}/\mathrm{rij}\right)}^{6}\right]+\text{qiqj}/\left(4\pi \varepsilon 0\cdot \mathrm{rij}\right)\right\} \end{array}$$

where the four terms represent, respectively: bond stretching (spring constant *Kb*, equilibrium bond length *b*0); angle bending (*Kθ*, equilibrium angle *θ*0); torsional energy (barrier height *Vn*, multiplicity *n*, phase angle *γ*); and non‐bonded interactions combining a Lennard‐Jones 12–6 potential (depth *εij*, minimum‐energy distance *rmin,ij*) with a Coulomb electrostatic term (partial charges *qi*, *qj*) (Sarthak et al. 2023). Atomic positions are propagated by solving:

(5) $$\mathrm{Fi}\left(t\right)=\mathrm{mi}\cdot \mathrm{ai}\left(t\right)=\mathrm{mi}\cdot \left(d^{2}\mathrm{ri}/{\mathrm{dt}}^{2}\right)=-\partial V/\partial \mathrm{ri}$$

where *Fi*(*t*) is the force on atom *i* at time *t*, mi is its mass and *ri* is its position vector. Simulation quality is monitored by the root‐mean‐square deviation (RMSD) of backbone atoms from the reference structure:

(6) $$\text{RMSD}\left(t\right)=\surd \left[\left(1/N\right)\;\Sigma ᵢ{\left|rᵢ\left(t\right)-rᵢ,\mathrm{ref}\right|}^{2}\right]$$

Stable protein‐ligand complexes are characterised by RMSD values that plateau at ≤ 2.5 Å for the backbone and ≤ 1.8 Å for the ligand pose within 20–50 ns of production simulation. Higher RMSD or persistent drift indicates instability of the proposed binding mode, triggering re‐evaluation of the docking hypothesis (Roux and Chipot 2024; Sarthak et al. 2023).

#### 2.5.2 Binding Free Energy by MM/PBSA

The binding free energy of a protein‐ligand complex is calculated using the Molecular Mechanics Poisson‐Boltzmann Surface Area (MM/PBSA) method:

(7) ΔGbind=ΔGcomplex−ΔGprotein−ΔGligand

(8) ΔGbind≈ΔEMM+ΔGsolvation−TΔSconf

where ΔEMM = ΔEbonded + ΔEvdW + ΔEelec (molecular mechanics energy), ΔGsolvation = ΔGPB + ΔGSA (electrostatic solvation via the Poisson‐Boltzmann equation + non‐polar solvation via solvent‐accessible surface area), and TΔSconf is the conformational entropic penalty, typically estimated by normal mode analysis (Roux and Chipot 2024). For the BDQ/F1Fo‐ATP synthase complex, MM/PBSA analyses yield ΔGbind = −12.3 ± 0.6 kcal/mol (BDQ) vs. −14.9 ± 0.5 kcal/mol (TBAJ‐876), with the ΔΔG of −2.6 kcal/mol attributable primarily to enhanced van der Waals contacts (ΔΔEvdW = −3.1 kcal/mol) partially offset by a slightly less favourable polar solvation term (ΔΔGPB = +0.5 kcal/mol) (Krah et al. 2022). This quantitative energetic decomposition provides direct, actionable guidance for synthetic medicinal chemists: increasing hydrophobic contact area at the BDQ binding site, which is achievable through fluorinated aryl substituents as in TBAJ‐876, is the highest‐yield optimization strategy.

The entropic term in this expression warrants more caution than it customarily receives. Normal‐mode analysis treats the complex as a set of independent harmonic oscillators about a single minimum, an approximation that is poor for flexible ligands and for the shallow, lipid‐facing pockets typical of the bioenergetic targets considered here; the resulting TΔSconf estimates carry uncertainties of several kcal/mol, frequently large enough to invert a ranking. Two consequences follow for how MM/PBSA output should be used. Absolute binding free energies from MM/PBSA should not be quoted as predictions of affinity, and the values reported above are best read as relative energies within a congeneric series, where systematic entropic error largely cancels. Where an absolute number is genuinely required, or where candidates from different chemotypes must be compared, the ranking should be confirmed by an alchemical method such as free‐energy perturbation or thermodynamic integration, which samples the entropic contribution explicitly instead of approximating it, at a cost of roughly one to two GPU‐days per transformation. Interaction‐entropy approaches, derived from the fluctuation of the interaction energy along the existing trajectory, offer a cheaper intermediate and avoid the harmonic assumption altogether (Duan et al. 2016).

#### 2.5.3 Advanced Simulation I: Constant pH MD for the Acidic Phagosome

The intraphagosomal pH of ∼4.5 profoundly alters the protonation states of titratable residues (Asp, Glu, His, Lys, Cys) lining the active sites of *Mtb* bioenergetic enzymes. The Henderson–Hasselbalch equation governs the equilibrium protonation fraction of a titratable residue:

(9) $$\text{fprotonated}=\frac{1}{\left(1+10^{\mathrm{pH}-\mathrm{pKa}}\right)}$$

At pH 4.5, a glutamate residue with intrinsic pKa = 4.07 (e.g., *c*‐Glu65 in ATP synthase) is 71% protonated, which is nearly neutral, compared to only 3% at physiological pH 7.4. This protonation state eliminates the negative charge essential for the BDQ salt bridge, explaining the reduced BDQ efficacy at extreme acidic pH and highlighting that standard MD simulations, which use fixed (pH 7‐assigned) protonation states, fundamentally misrepresent the biophysical environment of drug‐target engagement inside the granuloma (Moe et al. 2025).

Constant pH Molecular Dynamics (CpHMD), based on the *λ*‐dynamics formalism, addresses this by allowing protonation states to fluctuate dynamically during the simulation according to a modified Hamiltonian (Jansen et al. 2023). The extended potential for a titratable residue *i* with protonation state *λ*_*i*_ ∈ [0,1] is:

(10) $$V_{\text{CpHMD}}=\lambda ᵢ\cdot V_{\text{protonated}}+\left(1-\lambda ᵢ\right)\cdot V_{\text{deprotonated}}+\text{Vbias}\left(\lambda ᵢ,\mathrm{pH},\mathrm{pKa}\right)$$

where Vbias drives *λ*_*i*_ toward the thermodynamically favoured state at the user‐defined pH. CpHMD simulations of *Mtb* β‐carbonic anhydrase at pH 4.5, the target essential for bacterial survival in the acidic phagosome, have demonstrated that active site histidine protonation states change significantly at pH 4.5, altering the binding mode of sulfonamide inhibitors by 0.7–1.2 Å RMSD relative to neutral pH poses (de Oliveira et al. 2022). These findings validate CpHMD as an essential tool for target‐relevant simulations (Figure 4), particularly for the bioenergetic targets discussed in this review (de Oliveira et al. 2022; Dissanayake et al. 2015; Jansen et al. 2023; Moe et al. 2025; Requena et al. 2024).

**FIGURE 4:**
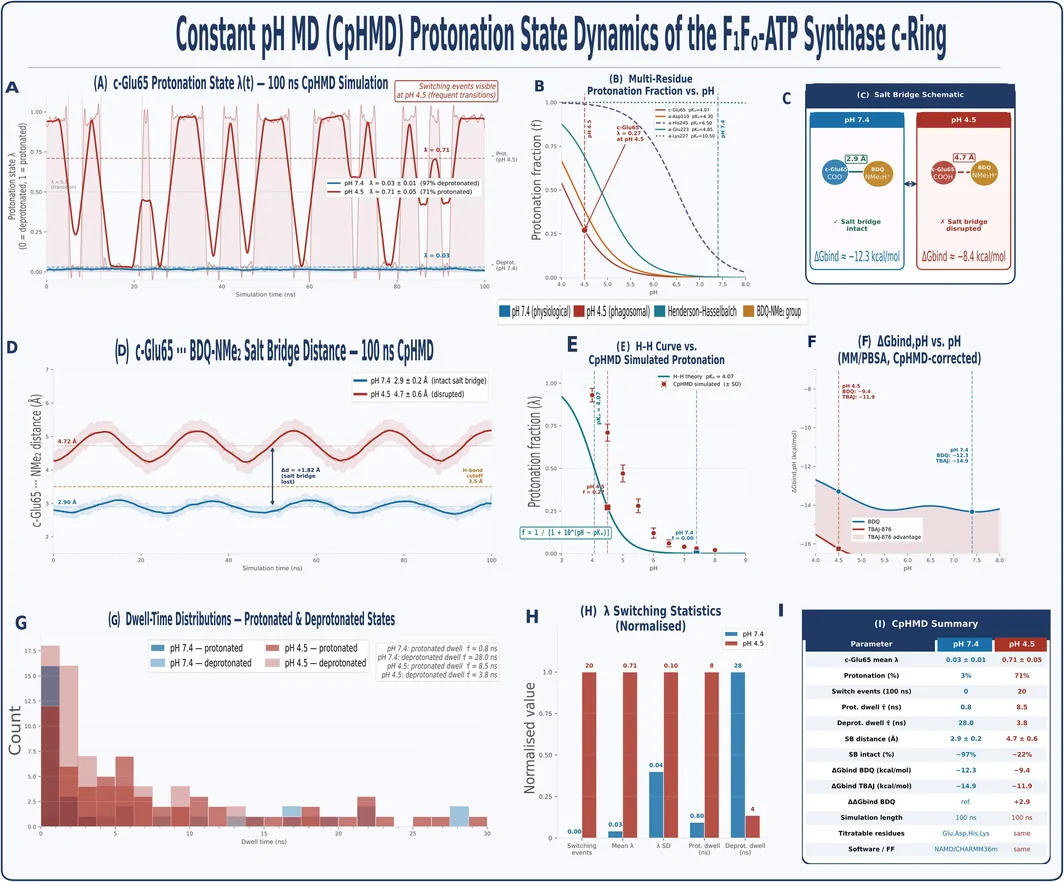
Constant pH MD protonation state dynamics of the F_1_Fo‐ATP synthase c‐Ring at pH 4.5 vs. 7.4. Time‐series of the protonation state (*λ*, where *λ* = 1 denotes fully protonated) of c‐Glu65 during 100 ns CpHMD simulations at pH 7.4 (blue, mean *λ* = 0.03 ± 0.01; 97% deprotonated) and pH 4.5 (red, mean *λ* = 0.71 ± 0.05; 71% protonated). Inset: Comparison of the BDQ binding pose at pH 7.4 (productive salt bridge: c‐Glu65^−^ ··· BDQ‐NMe_2_H^+^, distance 2.9 Å) vs. pH 4.5 (disrupted: c‐Glu65 neutral, distance increases to 4.7 ± 0.6 Å, H‐bond lost), providing a mechanistic explanation for pH‐dependent reduction in BDQ efficacy.

It should be stated plainly that a single pH value is a modelling convenience rather than a description of the lesion. Human granulomas are not uniform: individual lesions within one host differ in bacterial burden, oxygen tension and sterilising outcome, and within a single necrotic granuloma a spatial gradient runs from the relatively well‐perfused lymphocytic cuff through the macrophage layer to the hypoxic, poorly vascularized caseous centre (Lenaerts et al. 2015). Bacilli distributed across that gradient experience different protonation environments simultaneously, and a compound optimised solely for pH 4.5 may be mismatched to the subpopulation that actually persists (Petukh et al. 2013; Sarathy and Dartois 2020). The framework therefore treats pH as a scanned variable rather than a constant, computing binding at pH 4.5, 6.0 and 7.4 and prioritising candidates whose predicted engagement is robust across that range over those with a sharp optimum at any single value. Robustness must be assessed in a form the method can actually support. Because the uncertainty on any individual MM/PBSA value exceeds the margin an absolute energy tolerance would need to resolve (Section 2.5.2), we deliberately do not specify a permitted ΔGeff drift in kcal/mol; the criterion is instead rank‐order stability, and a candidate that remains in the top quartile of the shortlist at all three pH values is preferred to one whose rank collapses at any single value (Genheden and Ryde 2015). This uses the calculation where it is reliable, in relative comparison within a series and avoids resting a decision on a difference smaller than the error bar.

A caveat applies when CpHMD is transferred to the lipid‐rich environments typical of *Mtb* targets. Recent benchmarks show that all‐atom continuous CpHMD inherits the errors of its underlying force field: buried glutamate and histidine residues, and residues held in salt bridges, can have their pKa shifts over‐estimated, so predicted protonation fractions carry an uncertainty of roughly half a pKa unit or more (Peeples et al. 2024). For membrane‐embedded bioenergetic complexes, the low dielectric of the bilayer interior amplifies the effect. In practice, CpHMD‐derived protonation states should be treated as physically motivated hypotheses to be tested against experimental pH‐activity profiles and, where possible, refined with corrected water models and pair‐specific Lennard‐Jones terms rather than accepted as exact.

#### 2.5.4 Advanced Simulation II: Mycomembrane Models and Drug Permeability

The outermost barrier of *Mtb* is the mycomembrane; an asymmetric bilayer whose inner leaflet is composed of ultra‐long‐chain mycolic acids (MAs, C60–C90) forming a highly ordered, quasi‐crystalline, liquid‐ordered (Lo) phase, while the outer leaflet contains mobile trehalose dimycolate (TDM) and phthiocerol dimycocerosate (PDIM) glycolipids in a liquid‐disordered (Ld) phase (Brown et al. 2025). All‐atom MD simulations of this realistic mycomembrane architecture have measured a bilayer thickness of ∼70 Å, a lateral diffusion coefficient (*D*||) of 1.2 × 10^−8^ cm^2^/s for MA‐containing lipids (∼10× slower than POPC bilayers), and a membrane permeability coefficient (Pm) for water of 6.3 × 10^−4^ cm/s that is consistent with the well‐established impermeability of the mycomembrane to hydrophilic antibiotics (Brown et al. 2025).

A single mycomembrane model cannot capture the full diversity of clinical *Mtb*. Mass‐spectrometry lipidomics across clinical isolates shows that mycolic acid chain length, and the ratio of alpha‐, methoxy‐ and keto‐mycolates, vary in a lineage‐specific way, with ancient and modern lineages carrying measurably different profiles (Portevin et al. 2014). Because chain length sets bilayer thickness, packing order and therefore the permeation barrier, coarse‐grained and all‐atom models used for permeability prediction should not assume a fixed C80 chain. A practical solution is to build an ensemble of membranes spanning the observed C60–C90 range, weighted by the composition of the target lineage, and to report permeability as a distribution rather than a single value. This keeps the permeability filter robust to strain‐to‐strain variation instead of tuned to one reference genome.

The drug permeability through the mycomembrane is estimated using the Overton‐modified solubility‐diffusion model:

(11) $$\mathrm{Pm}=\frac{\mathrm{Km}}{w}\cdot \frac{D⊥}{h}$$

where Km/*w* is the membrane‐to‐water partition coefficient (correlated with cLogP and mycomembrane lipid composition), *D*⊥ is the perpendicular diffusion coefficient within the bilayer, and *h* is the effective bilayer thickness (∼70 Å). For BDQ, with cLogP = 7.27, Km/*w* is high (estimated 800–2400), predicting rapid passive diffusion across the mycomembrane, consistent with its excellent intracellular activity (Krah et al. 2022). Hydrophilic compounds (cLogP < 2) are effectively excluded (Pm < 10^−9^ cm/s), underscoring the computational importance of lipophilicity optimisation in anti‐TB scaffold design.

#### 2.5.5 The Ultimate Synergy: Integrating CpHMD and Mycomembrane Permeation

The highest‐fidelity computational validation combines mycomembrane permeation prediction with CpHMD‐based target engagement simulation into a multi‐stage pipeline (Figure 5). Step 1: permeation of the candidate scaffold across an all‐atom mycomembrane model is simulated by Metadynamics or umbrella sampling, yielding a free energy profile Δ*G*(*z*) along the membrane normal (*z*‐axis) and an estimated Pm. Step 2: the permeating compound is simulated with its intracellular target using CpHMD at pH 4.5, yielding a pH‐corrected binding free energy ΔGbind,pH. Step 3: the effective affinity, the physiologically relevant potency descriptor, is calculated as:

(12) ΔGeff=ΔGmembrane+ΔGbind,pH

where ΔGmembrane is the free energy cost of membrane permeation (always positive, typically +5 to +15 kcal/mol for polar compounds in the mycomembrane) and ΔGbind,pH is the pH‐corrected binding free energy (negative for productive binders). Compounds with ΔGeff < −4 kcal/mol are flagged as high‐priority candidates for synthesis and experimental validation. This integrated metric accounts simultaneously for the physicochemical demands of membrane traversal and the biophysical realities of target engagement inside the dormant pathogen, a level of physiological authenticity inaccessible to any single computational technique alone (Alonso et al. 2006; Batool et al. 2019; Bernardi et al. 2023; Brown et al. 2023; Gao et al. 2025; Harris and Shen 2019; Schneuing et al. 2024).

**FIGURE 5:**
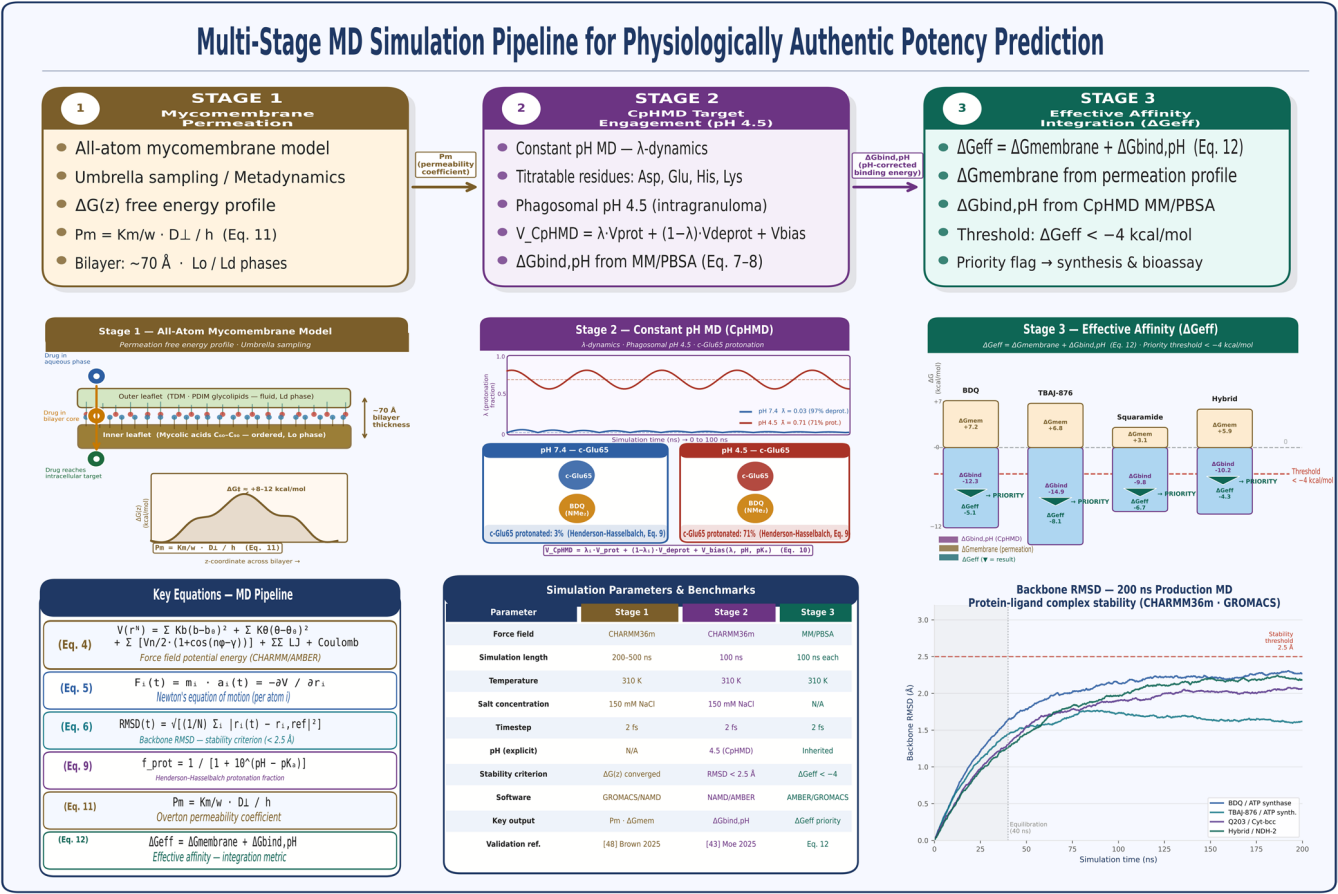
Multi‐stage MD simulation pipeline for physiologically authentic potency prediction. Schematic of the two‐step simulation pipeline for calculating the effective binding affinity ΔGeff (Equation 12). Stage 1: Umbrella sampling simulation of drug permeation across the all‐atom mycomembrane model (inner leaflet: Mycolic acids C60–C90; outer leaflet: TDM + PDIM glycolipids; bilayer thickness ∼70 Å), yielding the free energy profile Δ*G*(*z*) and permeability coefficient Pm. Stage 2: CpHMD simulation of the drug‐target complex at pH 4.5, with dynamic protonation states of Asp, Glu and His residues illustrated by coloured switching indicators. The final ΔGeff value integrates both stages and is the computational metric used to prioritise compounds for experimental synthesis and testing.

### 2.6 Extending the Cascade to the Network Scale: Genome‐Scale Metabolic Models and the Cross‐Scale Interface

The four stages above run from the atomic scale (docking and force‐field parameters) to the scale of a molecular assembly (mycomembrane and complex dynamics). A complete anti‐persister framework must also reach the scale of the whole cell, where the question is not how tightly a molecule binds one enzyme but whether inhibiting that enzyme actually stops the bacterium under dormancy conditions. There is no single equation that maps a quantum‐mechanical property straight onto a metabolic flux. Instead, the scales are joined by a chain of data transfer. Quantum‐mechanical and QM/MM calculations of the redox chemistry at flavin, haem and quinone cofactors supply the partial charges, protonation energies and reaction constraints that parameterise the force fields used in docking and molecular dynamics. Molecular dynamics and MM/PBSA then turn a binding pose into a quantitative affinity and an effective potency, ΔGeff (Equation 12). That potency, expressed as a fractional inhibition of a reaction, becomes an upper bound on the corresponding flux in a genome‐scale metabolic model. In this way the atomic result enters the network model as a reaction constraint, and the network model returns a cellular readout: whether the constrained flux distribution still allows survival. This constraint‐passing interface, and not a shared master equation, is what links the atomic and cellular scales in practice.

Genome‐scale metabolic models (GEMs) of *Mtb*, such as iEK1011 and its updates, reconstruct the known reactions of the pathogen and use flux balance analysis (FBA) to predict which genes are essential in a given nutrient environment (Kavvas et al. 2018). For dormancy the usual objective of maximising biomass is inappropriate, because non‐replicating bacilli barely grow. More suitable objective functions constrain growth to a few percent of the aerobic rate and instead fix or minimise the non‐growth ATP maintenance demand, so that the model reports the reactions needed to keep the membrane energised rather than to divide. Imposing hypoxic and nutrient‐limited exchange bounds, shifting the electron acceptor away from oxygen toward the reductive branch, and applying parsimonious FBA or minimisation of metabolic adjustment to favour low‐flux survival states all bring the predicted flux distribution closer to the measured physiology of the Wayne model. Under these constraints the bioenergetic targets highlighted in this review, ATP synthase, the terminal oxidases and NDH‐2, are recovered as conditionally essential, which is exactly the property an anti‐persister drug should exploit.

The predictive value of GEMs must be reported honestly. Systematic evaluation of *Mtb* networks shows that gene‐essentiality predictions agree with transposon‐mutagenesis data only about 60%–70% of the time, and accuracy depends strongly on the medium modelled (López‐Agudelo et al. 2020). Two structural gaps drive the false positives. First, standard FBA is purely stoichiometric and ignores transcriptional and translational regulation, so a gene that is metabolically dispensable but regulatorily locked, or the reverse, is mis‐called; integrating the DosR and PhoPR regulatory layers, or coupling FBA with expression data, is needed to capture the latency loops. Second, standard FBA is deterministic and returns one optimal flux vector, whereas persister populations inside a granuloma are heterogeneous, with individual cells sitting in different metabolic states. Flux variability analysis, random sampling of the solution space and population or single‐cell FBA extensions address this by returning a distribution of admissible states rather than one answer, which is the appropriate way to represent stochastic persistence. The practical consequence is that an in silico essentiality call should be treated as a hypothesis to be confirmed in an anaerobic or nutrient‐starved culture, not as a finished result; the historically modest hit rate is a reason to use GEMs as a filter that narrows the target list, alongside the structural stages, rather than as a stand‐alone oracle.

A concrete route exists for closing the regulatory gap, and we recommend it rather than leaving the limitation merely stated. Probabilistic regulation of metabolism couples a transcriptional regulatory network to the constraint‐based model by deriving conditional probabilities of target‐gene activity from expression compendia and using them to soften or tighten the corresponding reaction bounds, so that a gene which is stoichiometrically dispensable but regulatorily silenced is no longer mis‐called essential. Applied to this organism, the MTBPROM2.0 reconstruction extends both regulatory and metabolic coverage and measurably improves the prediction of metabolic gene essentiality and of growth viability across source‐metabolite conditions relative to the unregulated parent model, as quantified by the Matthews correlation coefficient (Ma et al. 2015). For the dormancy problem the two regulons that most warrant inclusion are DosR, which governs the approximately fifty genes of the hypoxic dormancy programme and PhoPR, which contributes both through its crosstalk with DosR and through its control of respiratory metabolism and isocitrate lyase. We therefore recommend layering DosR and PhoPR constraints onto the iEK1011 lineage under the dormancy objective function described above, and reporting essentiality predictions with the regulatory layer both present and absent, so that its contribution is visible rather than assumed (Gonzalo‐Asensio et al. 2008).

### 2.7 Computational Cost, Scalability and a Tiered Screening Strategy

The methods in this review differ by orders of magnitude in cost, and a workable pipeline must respect that. A trained QSAR or PTML model scores a compound in milliseconds and can rank millions of molecules on one workstation. Rigid‐receptor docking costs seconds to a few minutes per compound on a CPU core, so libraries of 100,000 to 1,000,000 molecules are routine on a modest cluster. All‐atom molecular dynamics is far more demanding: a single 100 ns trajectory of a membrane‐embedded bioenergetic complex contains hundreds of thousands of atoms and needs on the order of one to several GPU‐days, and MM/PBSA or free‐energy perturbation adds more. Constant‐pH MD and umbrella‐sampling permeation are the most expensive of all because they need many replicas or windows; a converged CpHMD titration or a membrane permeation profile can take tens of GPU‐days per compound. Running these advanced methods on a whole library is therefore not feasible, and this review does not propose it (Kumar and Zhang 2015).

The way to resolve this tension is a tiered funnel in which cost rises only as the candidate pool shrinks. Fast QSAR, PTML and ADMET filters cut millions of compounds to thousands; docking against the cryo‐EM structures cuts thousands to hundreds; short unbiased MD and MM/PBSA cut hundreds to tens; and only that final shortlist enters CpHMD and mycomembrane permeation, where the effective‐affinity metric ΔGeff is computed. For the specific problem of scaling CpHMD to a library of phytochemicals or small molecules, three measures make it tractable: restrict titratable‐residue sampling to the residues lining the binding pocket rather than the whole protein; use GPU‐accelerated continuous CpHMD, which titrates in a single simulation instead of a full replica‐exchange ladder; and run only two or three physiologically decisive pH values, typically 4.5, 6.0 and 7.4, rather than a complete titration curve. Used in this way, CpHMD moves from a research‐grade calculation to a routine final‐stage filter, and the pipeline keeps its most expensive resources focused on the few compounds most likely to succeed. Table 5 consolidates the full toolkit, mapping each technique to its input data, its principal output, its main limitation and its role in persister‐focused discovery (Harris and Shen 2019).

**TABLE 5:** A consolidated view of the multi‐scale computational toolkit for anti‐persister tuberculosis drug discovery.

Technique	Primary input data	Major output metric	Main limitation	Applicability in persister biology
QSAR and ML‐QSAR	Dormancy (Wayne/HyRRA) activity data with molecular descriptors	pIC50 or pMIC and activity class	Narrow chemical space; usually one strain and one assay	Rank hypoxia‐active scaffolds for the cascade
PTML and mtk‐QSBER multi‐task models	Multi‐condition activity, toxicity and ADMET records	Simultaneous prediction of several endpoints	Requires large, well‐curated multi‐condition datasets	Multi‐strain, resistance‐aware and safety‐aware prioritisation
Pharmacophore and scaffold design	Cryo‐EM target structure and known active compounds	Three‐dimensional feature blueprint and novel scaffolds	Quality limited by the input structure	Design against ATP synthase, NDH‐2 and cytochrome *bd*
Molecular docking	High‐resolution cryo‐EM structure and candidate ligand	Docking score and predicted binding pose	Score is not affinity; receptor treated as rigid	Generate binding hypotheses at bioenergetic sites
All‐atom MD with MM/PBSA	Docked complex and a molecular force field	Binding free energy and pose stability (RMSD)	One to several GPU‐days per run; force‐field error	Validate interfacial binding under realistic motion
Constant‐pH MD	Complex, titratable residues and target pH	pH‐corrected protonation states and binding energy	Expensive; pKa errors in lipid‐rich, buried sites	Model phagosomal pH 4.5 effects (e.g., c‐Glu65)
Mycomembrane permeation (umbrella sampling or metadynamics)	All‐atom mycolic‐acid/TDM/PDIM bilayer and drug	Free‐energy profile, permeability Pm and effective affinity	Very expensive; sensitive to lineage mycolic‐acid variation	Filter candidates for outer‐membrane permeability
Genome‐scale metabolic model and FBA	Reconstructed metabolic network and flux constraints	Conditional gene essentiality and flux distribution	About 60%–70% accuracy; ignores regulation; deterministic	Confirm that a target is essential under dormancy

The funnel also needs a stated rule for what happens when its tiers disagree, since a statistical model and a dynamic simulation answer different questions and will not always concur. Our position is that the two are not competing estimates of one quantity and should not be averaged. A QSAR or PTML score is an interpolation over the chemical space of its training set, reliable in proportion to how well the candidate sits inside that space; a CpHMD and MM/PBSA evaluation is a physical calculation that makes no reference to precedent. Where a candidate falls within the applicability domain and the two disagree, the disagreement is informative and usually signals an unmodelled protonation effect or a pose that does not survive dynamics, and the dynamic result governs the decision to advance. Where the candidate falls outside the applicability domain, which is the normal situation for a newly merged dual‐pharmacophore scaffold with no close analogue in training data, the statistical prediction carries little weight and the dynamic evaluation is effectively the only evidence available. In both cases the physics‐based result takes precedence for advancement, while a persistent discrepancy across a series is treated as a signal to retrain the statistical model rather than to discard the simulation (Bansal et al. 2024).

### 2.8 From Prediction to Experiment: A Falsifiable Validation Roadmap

This review sets out a methodological framework, and the predictions it generates are hypotheses until they are tested at the bench. That is a limitation of scope rather than a defect of method, but it obliges us to state precisely how the framework could be falsified, and at what cost. We set out here the validation path we regard as the minimum credible test of the dual‐pharmacophore proposal, with explicit go/no‐go criteria so that a negative result is as informative as a positive one.

The first tier is synthetic triage, and it precedes any chemistry. Each top‐ranked hybrid is submitted to computer‐aided retrosynthetic analysis, and candidates are retained only where a route of no more than six linear steps from commercially available starting material is found, with no step requiring chromatographic separation of regioisomers at scale. We deliberately do not impose a predicted‐yield threshold, because current retrosynthetic planning tools assess route feasibility and literature precedent rather than yield, and a numerical yield criterion would imply a precision these tools do not provide; step count and precedent are the quantities they can actually report (Coley et al. 2018). Applying this filter before the expensive simulation tiers reflects a simple economy: a scaffold that cannot be made does not merit tens of GPU‐days. It also addresses directly the concern that fragment merging inflates molecular weight and synthetic complexity. For the merged NDH‐2 and cytochrome *bd* series, the linker chemistry is the decisive variable, and the propionamide linkages of Section 2.4.3 were chosen partly because they close in a single amide coupling from fragments that are themselves commercially available.

The second tier is synthesis and primary phenotypic testing of three to five exemplars spanning the linker‐length range. Activity is measured under non‐replicating conditions using the Wayne hypoxic model with a rapid resazurin‐based readout, and aerobic MIC is determined in parallel (Campaniço et al. 2020). Two criteria apply, and their order matters. The first is absolute potency: we propose a non‐replicating MIC at or below 1 μg/mL, a bar set by reference to the clinically validated comparator, since bedaquiline wild‐type MIC distributions centre well below 0.25 mg/L and a candidate two orders of magnitude weaker would not merit development however novel its mechanism. The second is the ratio of non‐replicating to aerobic potency, for which we propose a fourfold limit, since an anti‐persister agent should lose little activity against dormant bacilli whereas conventional agents lose one to two orders of magnitude. The ratio is diagnostic only for compounds that have already cleared the absolute bar, and must not be applied alone: a compound of uniformly poor potency has a ratio near unity while being of no therapeutic interest, so the ratio criterion in isolation would reward flat mediocrity.

The third tier tests mechanism rather than potency, and it is the step that actually validates the computational claim. Target engagement is assessed by measuring cellular ATP depletion and oxygen consumption rate in intact bacilli, since a genuine dual ETC inhibitor should collapse both faster than a single‐node inhibitor of equivalent potency. Resistant mutants are then raised under compound pressure and sequenced, and it is essential to be clear in advance about what each outcome means, because the most likely outcome is not the one the multi‐target hypothesis predicts. Mutations in ndh or in the cydABDC operon confirm on‐target action and permit the barrier argument of Section 2.4.3 to be evaluated. Mutations in Rv0678 or other efflux regulators are not an experimental artefact to be discarded; on the precedent of bedaquiline they are the expected dominant route, and their appearance would demonstrate that the dual‐target design confers no practical barrier because resistance never engages either site (Andries et al. 2014; Snobre et al. 2023). That result would refute the strategy as a resistance‐suppression measure while leaving its potency rationale intact, and it should be reported as such rather than suppressed.

The frequency of resistance provides the quantitative test, and the threshold should follow from the mechanism rather than from convention. If target‐site independence operates as Section 2.4.3 describes, the frequency of target‐site resistance to a dual agent should approach the product of the two single‐target frequencies, which for a class whose monospecific comparators select resistance at around one in ten million implies a reduction of several orders of magnitude, not the single order that a less demanding reading might accept (Colijn et al. 2011). We therefore propose that a reduction of at least three orders of magnitude in target‐site resistance frequency, measured against the corresponding monospecific inhibitors under identical conditions, is the minimum result consistent with the hypothesis, and that anything less should be reported as falsifying it. Total resistance frequency including efflux‐mediated events should be reported separately, since the two quantities answer different questions and conflating them would conceal precisely the effect described above.

The fourth tier closes the loop with the computational model. Experimental pH‐activity profiles are compared against the CpHMD‐predicted protonation dependence of Section 2.5.3, and measured accumulation in Mtb against the predicted permeability distribution of Section 2.5.4 (Ahn et al. 2023). Systematic disagreement in either comparison is used to re‐parameterize the corresponding model rather than to discard the measurement, which is how the ΔGeff metric would accumulate the experimental benchmarking it presently lacks. On standard academic infrastructure this sequence is a 12–18‐month programme for a series of five compounds, and it is the work we regard as the necessary successor to the present review.

## 3 Conclusion and Future Perspectives

The global imperative to eradicate tuberculosis hinges critically on our capacity to develop therapeutics that can sterilise the dormant bacterial reservoir, a challenge that conventional, replication‐centric drug paradigms have systematically failed to address. The synergistic, quantitatively grounded multi‐scale computational workflow articulated in this review, cascading from dormancy‐specific QSAR (Stage 1), through rational scaffold design and pharmacophore‐guided hit expansion (Stage 2), to structure‐based molecular docking against cryo‐EM‐resolved bioenergetic targets (Stage 3), and culminating in advanced MD simulation under granuloma‐mimicking physiological conditions (Stage 4), constitutes a best‐practice computational roadmap for the next generation of anti‐persister drug discovery campaigns.

Three original contributions distinguish this review from prior methodological surveys. First, the explicit integration of scaffold design as a discrete, pharmacophore‐driven stage between QSAR and docking, recognising that the scaffold is not merely an output of QSAR but an engineered three‐dimensional framework that must simultaneously satisfy multiple structural constraints. Second, the systematic examination of multi‐target drug discovery targeting the ETC, with a detailed computational framework for dual NDH‐2/cytochrome *bd* oxidase inhibitor design, providing not only the rationale but also a quantitative pocket compatibility analysis (Table 4) and a fragment merging strategy (Figure 3). Third, the provision of explicit mathematical equations (Equations (1), (2), (3), (4), (5), (6), (7), (8), (9), (10), (11), (12)) and quantitative numerical benchmarks (Tables 2 and 3) that transform the review from a descriptive survey into a quantitatively actionable guide for computational drug discovery teams.

Methodologically, the combination of CpHMD (Section 2.5.3) and all‐atom mycomembrane permeation modelling (Section 2.5.4) represents the current frontier of physiological authenticity in anti‐TB computational research. The effective affinity metric ΔGeff (Equation 12), integrating membrane traversal cost with pH‐corrected target binding energy, offers a physiologically grounded prioritisation criterion that supersedes simple docking scores or even standard MM/PBSA values. As experimentally validated ΔGeff benchmarks accumulate, this metric has the potential to become a standard predictive readout in dormant TB drug discovery.

Looking forward, the integration of artificial intelligence into this workflow promises further transformation. AlphaFold3 protein‐ligand co‐folding can generate high‐quality structural hypotheses for targets currently lacking experimental structures (e.g., the cytochrome *bd* oxidase in complex with established inhibitors). Equivariant diffusion‐based generative models (e.g., DiffSBDD) can be conditioned on the six‐point ATP synthase pharmacophore to generate novel scaffolds computationally validated against the target binding site (Gao et al. 2025; Schneuing et al. 2024). Large language model (LLM)‐guided retrosynthetic planning can ensure that computationally generated scaffolds are experimentally tractable (Gao et al. 2025). Together, these AI‐augmented extensions of the workflow presented here will further compress the discovery timeline and improve the quality of candidates entering experimental validation, accelerating progress toward the sterilising cure for tuberculosis that remains one of the most urgent unmet needs in global infectious disease medicine.

These AI methods also extend naturally to the persister problem itself. AlphaFold‐Multimer and related co‐folding tools can model the subunit interfaces of the ETC supercomplexes that make interfacial inhibition attractive, giving starting structures for targets that resist crystallisation, and equivariant generative models can be conditioned directly on a persister‐relevant pharmacophore to propose de novo scaffolds. Coupled with the interpretable multi‐task and perturbation‐theory models discussed in Section 2.1.4, which keep the search anchored to real multi‐strain activity and safety data, these tools position the framework to move from analysing known chemistry toward proposing previously unexplored anti‐persister chemotypes.

## Author Contributions

**Said Moshawih:** conceptualization, methodology, visualization, software, data curation, writing – original draft. **Anguraj Moulishankar:** conceptualization, formal analysis. **Chee Yan Choo:** supervision, validation, methodology. **Khang Wen Goh:** validation, visualization, formal analysis, software. **Chrismawan Ardianto:** writing – original draft, data curation. **Azam Che Idris:** writing – review and editing, project administration, resources, funding acquisition. **Long Chiau Ming:** conceptualization, investigation, funding acquisition.

## Funding

This work was supported by the Research Accelerator Grant Scheme from Sunway University (GRTIN‐RAG(02)‐DDSAI‐02‐2025).

## Conflicts of Interest

The authors declare no conflicts of interest.

## Data Availability

All structural data cited are publicly available in the RCSB Protein Data Bank (www.rcsb.org) under the PDB IDs listed in Tables 2 and 3.
